# Boolean Network Model Predicts Cell Cycle Sequence of Fission Yeast

**DOI:** 10.1371/journal.pone.0001672

**Published:** 2008-02-27

**Authors:** Maria I. Davidich, Stefan Bornholdt

**Affiliations:** Institut für Theoretische Physik, Universität Bremen, Bremen, Germany; IBM Thomas J. Watson Research Center, United States of America

## Abstract

A Boolean network model of the cell-cycle regulatory network of fission yeast (*Schizosaccharomyces Pombe*) is constructed solely on the basis of the known biochemical interaction topology. Simulating the model in the computer faithfully reproduces the known activity sequence of regulatory proteins along the cell cycle of the living cell. Contrary to existing differential equation models, no parameters enter the model except the structure of the regulatory circuitry. The dynamical properties of the model indicate that the biological dynamical sequence is robustly implemented in the regulatory network, with the biological stationary state G1 corresponding to the dominant attractor in state space, and with the biological regulatory sequence being a strongly attractive trajectory. Comparing the fission yeast cell-cycle model to a similar model of the corresponding network in *S. cerevisiae*, a remarkable difference in circuitry, as well as dynamics is observed. While the latter operates in a strongly damped mode, driven by external excitation, the *S. pombe* network represents an auto-excited system with external damping.

## Introduction

Predicting the dynamics of complex molecular networks that control living organisms is a central challenge of systems biology. While cell-wide, or organism-wide, models of genetic and molecular interactions appear well out of reach, predictive models of single pathways and small modular molecular networks of living cells have been studied with great success and are a matter of active research [1]–[4].

If the biochemical details of a chemical molecular network are known, an efficient yet detailed method for its simulation is to use chemical Monte-Carlo simulations [5], [6]. Less computationally costly, and perhaps the most commonly used approach to modeling biochemical pathways and networks, are differential equations which capture the underlying reaction kinetics in terms of rates and concentrations [7]. Such methods are highly developed today and are broadly applied to predictive dynamical modeling from single pathways to complex biochemical networks [8].

Such mathematical models contain detailed information about the time evolution of the system, which, in some circumstances, may be more than one is interested in. For many biological questions, the sole prediction of the sequential pattern of states of the central control circuit of a cell could advance our knowledge significantly, as may be the case in cell cycle progression, cell commitment (e.g. to apoptosis), and in stem cell control and differentiation. When we are interested in the path that a cell takes, the exact time course of the control circuit dynamics may not be needed, however, its modeling takes most of our efforts and often one needs to know large numbers of biochemical parameters that are not easily obtained [9], [10].

Indeed, recent research indicates that some molecular control networks are so robustly designed that timing is not a critical factor [11]. Vice versa, as a working hypothesis, this observation bears the chance for vastly simplified dynamical models for molecular networks, as soon as one drops the requirement for accurate reproduction of timing by the model, and just asks for the sequence of dynamical patterns of the network. Recent studies demonstrate, that such more simplified models indeed can reproduce the sequence of states in biological systems. For example, a class of discrete dynamical systems with binary states, mathematically similar to models used in artificial neural networks, has recently proven to predict specific sequence patterns of protein and gene activity as observed in living cells [12], [13].

Such models are in the mathematical tradition of random Boolean networks which, for decades, served as a simplistic analogy for how gene regulation networks could in principle work [14]. In these historical studies, dynamical properties of random networks of discrete dynamical elements were studied to derive possible properties of (the then hardly known) regulatory circuits [15]. In the new approach outlined above, however, similar mathematical elements now serve to simulate one specific biological control network of fully known circuitry. From a different perspective, they can be viewed as a further simplification of the differential equation approach [16]. Recent application of this model class to modeling real biological genetic circuits show that they can predict sequence patterns of protein and gene activity with much less input (e.g. parameters) to the model as the classical differential equations approach. Examples are models of the genetic network underlying flower development in *A. thaliana* [17]–[19], the cell-cycle networks of *S. cerevisiae* [13], the signal transduction network for abscisic acid induced stomatal closure [20], the mammalian cell-cycle [21], as well as gene regulatory networks determining embryonic segmentation in *D. melanogaster* [12], [21].

For example, the model by Albert and Othmer [12] of the segment polarity gene network in *D. melanogaster*, as well as the model by Li et al. [13] of the *S. cerevisiae* cell-cycle control network, yield accurate predictions of sequential events of the processes previously not obtained from such a simple model class. In these models, the dynamics can be viewed in terms of flow in the space of possible states of the network, converging towards so-called attractors, or fixed points, which here correspond to specific biological states. These attractors and their basins of attraction in state space mainly depend on the circuitry of the network, and their analysis yields further information about the robustness of the dynamics against errors or mutations.

How generic is this approach? In this article we address the question whether the approach of discrete dynamical network models is a more general method, namely whether constructing predictive dynamical models for regulation of proteins and genes from Boolean networks is a straightforward procedure that generalizes to other organisms. We choose the fission yeast (*Schizosaccharomyces Pombe*) cell-cycle as an example system that on the one hand is well understood in terms of established differential equation models, but on the other hand is markedly different from the above examples, as *S. cerevisiae*. The yeast *S. Pombe* has been sequenced in 1999 and has been used as a model organism only relatively recently [23]. Models exist [24], [25] that mathematically model the fission yeast cell-cycle with a common ODE (ordinary differential equation) approach. These are based on a set of differential equations for the biochemical concentrations that take part in the network and their change in time (and space). This approach allows to predict the dynamics of the fission yeast cell-cycle for the wild-type and some known mutant cells [10], [26].

We will in the following construct a discrete dynamical model for the fission yeast cell cycle network. An interesting question will be, how far we will get without considering parameters, as kinetic constants etc., that are a key ingredient of the existing models. We will base our model on the circuitry of the known biochemical network, only. Let us in the next section briefly review the fission yeast cell cycle network, then define our discrete dynamical model in the subsequent section. This is followed by a section reporting our results, and then we will compare our findings with a similar model of the budding yeast (*S. cerevisiae*) network and conclude with a discussion.

### The fission yeast cell cycle network

Let us briefly review the regulatory processes that control the cell cycle in *S. Pombe*. The full process of one cell division consists of four stages, named G1—S—G2—M. At the first stage (G1), the cell grows and, under specific conditions, commits to division. At the second stage (S), DNA is synthesized and chromosomes are replicated. This is followed by a “gap” stage G2. The final stage (M) corresponds to mitosis, in which chromosomes are separated and the cell divides itself. Eventually, after the M stage, the cell enters G1 again, thereby completing one cycle.

The biochemical reactions that form the network that controls the fission yeast cell-cycle have been studied in detail over the last years [25], [27]–[34]. The major role is played by a cyclin-dependent protein kinase complex Cdc2/Cdc13 with Tyr-15, a residue of Cdc2. When Tyr-15 is unphosphorylated, complex Cdc2/Cdc13 reaches high activity. This residue is inactive during the G2 phase, when Cdc2/Cdc13 is phosphorylated, and becomes active during the G2—M transition [25], [26]. We have two nodes, representing this complex: Cdc2/Cdc13 and Cdc2/Cdc13*. The first is responsible for the intermediate activity of the complex, when the residue Tyr-15 is in its inactive form. Cdc2/Cdc13* is an indicator of high activity of Cdc2/Cdc13, when Tyr-15 is unphosphorylated.

The other members that participate in the cell-cycle control can be attributed to two different classes. The first class consists of positive regulators of the kinase Cdc2/Cdc13: an indicator of mass of the cell, works as “Start”, “Start kinase” (SK), a group of Cdk/cyclin complexes (Cdc2 with Cig1, Cig2 and Puc1 cyclins), and the phosphatase Cdc25. A second class is composed of the antagonists of the complex Cdc2/Cdc13: Slp1, Rum1, Ste9, and the phosphatase PP [9].

We give a full compilation of the network of key-regulators of the fission yeast cell cycle network in Table 1, corresponding to our current knowledge as given in [9], [25], [26]. Also our translation into an interaction graph with activating and inhibiting links is given in the table, which is the starting point for our discrete dynamical network simulation of this network. Let us in the next section define the discrete dynamics that we will simulate on this graph.

**Table 1:** The rules of interaction of the main elements involved in the fission yeast cell cycle regulation.

Parent node	Daughter node	Rule of activation (comments)	Rule of inhibition (comments)
Start node	Starter Kinases (SK): Cdc2/Cig1, Cdc2/Cig2, Cdc2/Puc1	Start node works as an indicator of mass of the cell and activates Start Kinases (SK) Cdc2/Cig1, Cdc2/Cig2, Cdc2/Puc1, +1[9]	
SK	Ste9, Rum1		Phosphorylate, thereby inactivate, −1 [9], [25]
Cdc2/Cdc13	Cdc25	Cdc25 is phosphorylated thereby activated, +1 [9].	
Wee1, Mik1	Cdc2/Cdc13*		Phosphorylate, inactivating, −1 [9]
Rum1	Cdc2/Cdc13, Cdc2/Cdc13*		Binds and inhibits activity, −1 [9].
Cdc2/Cdc13	Rum1		Phosphorylates and thereby targets Rum1 for degradation. −1 [9], [25]
Ste9	Cdc2/Cdc13, Cdc2/Cdc13*		Labels Cdc13 for degradation [25], [9], −1.
Cdc2/Cdc13*	Slp1	Highly activated Cdc2/Cdc13* activates Slp1, [24], [9] +1.	
Slp1	Cdc2/Cdc13, Cdc2/Cdc13*		Promotes degradation of Cdc13, thereby the activity of Cdc2/Cdc13 drops −1 [9]
Slp1	PP	Activates, +1 [9]	
PP (Unknown phosphatase)	Ste9, Rum1, Wee1, Mik1	Activates Rum1, Ste9, and the tyrosine-modifying enzymes (Wee1, Mik1) [9], +1	
Cdc25	Cdc2/Cdc13*	Cdc25 reverses phosphorylation of Cdc2, thereby Cdc2/Cdc13* becomes active, +1 [9], [24]	
Cdc2/Cdc13	Ste9		inhibits −1 [24]
PP	Cdc25		inhibits −1 [9]
Cdc2/Cdc13	Wee1, Mik1		inhibits −1 [24]
Cdc2/Cdc13*	Rum1, Ste9		Inhibits −1 [24]

### A discrete dynamical model of the cell cycle network

**Figure 1:**
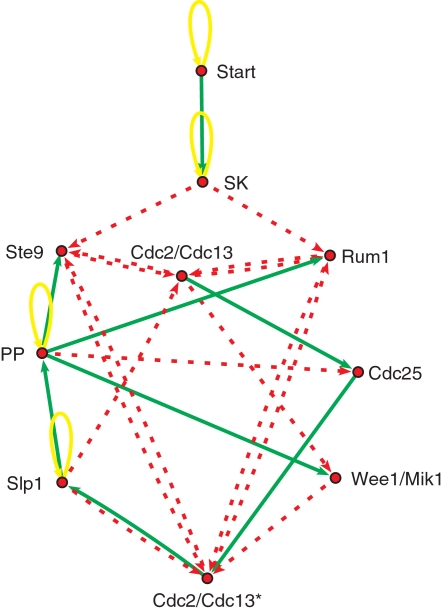
Network model. Network model of the fission yeast cell-cycle regulation. Nodes denote threshold functions (1), representing the switching behavior of regulatory proteins. Thresholds for the specific nodes are chosen as described in the text. Arrows represent interactions between proteins as defined in the interaction matrix *a_ij_* of the model (with *a_ij_ = +1* for green/solid arrows and *a_ij_ = −1* for red/dashed arrows).

We assume proteins to be the nodes of the network and assign a binary value *S_i_(t)*∈{0,1} to each node *i*, denoting whether the protein is present or not (due to different possible biochemical mechanisms, as, e.g., gene expression of a corresponding protein, or fast biochemical reactions as phosphorylization). The interactions between the nodes, as compiled in Table 1, are denoted as links, or arrows (see Figure 1). We do not quantify any interaction strength, except whether a link is present or not, and whether it is activating or inhibiting. Again, different biochemical mechanisms are subsumed under this simplified picture, as, e.g., transcriptional regulation, or faster enzymatic interactions.

The states of the nodes are updated (in parallel) in discrete time steps according to the following rule:
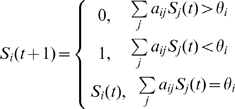
(1)where *a_ij_(t) = 1* for an activating interaction (green link) from node *j* to node *i*, *a_ij_(t) = −1* for an inhibiting (red) link from node *j* to node *i*, and *a_ij_(t) = 0* for no interaction at all. This definition follows closely the approach in [13]. *θ_i_* is a threshold of activation of node *i*, which is 0 for all nodes, except two explained below. The dramatic simplification step in constructing this model consists in not differentiating between absolute values of interaction strengths on the one hand, and not distinguishing between the different time scales of the biochemical interactions involved on the other. This corresponds to dropping all biochemical parameter values, time constants, as well as binding constants, from the differential equation models. As we will see below, dynamical models on networks can be built to be insensitive to these parameters, provided that the interaction topology has certain properties.

Two of the ten nodes included in the model exhibit a slightly different activation behavior, which we account for by a non-zero activation threshold. Cdc2/Cdc13*, the highly activated form of the complex Cdc2/Cdc13, has to be actively maintained by a positive regulatory signal, therefore *θ_i_* = 1 for this node. The second special rule is to add “self-activation” (corresponding to adding a negative activation threshold *θ_i_* = −1) to the node Cdc2/Cdc13, as it is otherwise not positively regulated. The biological motivation for this rule is the following. Cdc13 is constantly synthesized and after synthesis it immediately associates with Cdc2 [24]. Intracellular concentration of Cdc2 does not vary throughout the cell cycle [26]. Thereby, as soon as enemies are not active, Cdc2/Cdc13 is becoming active. A similar mechanism is implemented in the corresponding ODE model [24] in terms of an inhomogeneous differential equation for Cdc13_T_ with a heterogeneous exciting term k_1_M.

We also follow [13] by adding “self-degradation” (yellow loops) to those nodes that are not negatively regulated by others, representing the continuous degradation of proteins in the cell, which corresponds to *a_ii_(t) = −1*.

Nodes, that have the same function as, for example, Wee1/Mik1 and SK (Cdc2/Cig1, Cdc2/Cig2, Cdc2/Puc1) are joined together in a single node (see Figure 1), as it does not make a difference in the specific mathematical model dynamics considered here.

Finally let us define the initial condition of the model at the start of the simulation, which is chosen to correspond to the biological start condition with all nodes being in the OFF (inactive) state, except for the proteins Start, Ste9, Rum1, and Wee1/Mik1 [26].

## Results

### Simulation of the fission yeast cell cycle

Let us first consider the time evolution of the proteins of the dynamical model described above. We run the cell-cycle model by exciting the G1 stationary state with the cell size signal (“Start” node). This initiates a sequence of network activation states of proteins that, eventually, return to the G1 stationary state. The temporal evolution of the protein states is presented in Table 2, where one observes a sequence of states which exactly matches the corresponding biological time sequence in the cell-cycle control network, from the excited G1 state (START) through S and G2 to the M phase and finally back to the stationary G1 state. This is a remarkable observation as it is unlikely to occur by chance due to the size of the state space.

**Table 2:** Temporal evolution of protein states in the cell cycle network.

Time Step	Start	SK	Cdc2/Cdc13	Ste9	Rum1	Slp1	Cdc2/Cdc13*	Wee1 Mik1	Cdc25	PP	Phase	comments
1	1	0	0	1	1	0	0	1	0	0	START	Cdc2/Cdc13 dimers are inhibited, antagonists are active.
2	0	1	0	1	1	0	0	1	0	0	G1	SK are becoming active
3	0	0	0	0	0	0	0	1	0	0	G1/S	When Cdc2/Cdc13 and SK dimers switch off Rum1 and Ste9/APC, the cell passes ‘Start’ and DNA replication takes place, Cdc2/Cdc13 starts to accumulate
4	0	0	1	0	0	0	0	1	0	0	G2	Activity of Cdc2/Cdc13 achieves moderate level, which is enough for entering G2 phase but not mitosis, since Wee1/Mik1 inhibits the activity of residue Tyr-15 of Cdc2 (Cdc2/Cdc13* is not active)
5	0	0	1	0	0	0	0	0	1	0	G2	Moderate activity Cdc2/Cdc13 activates Cdc25
6	0	0	1	0	0	0	1	0	1	0	G2/M	Cdc25 reverses phosphorylation, removing the inhibiting phosphate group and activating Cdc2/Cdc13*
7	0	0	1	0	0	1	1	0	1	0	G2/M	Cdc2/Cdc13* reaches high activity level sufficient to activate Slp1/APC mitosis
8	0	0	0	0	0	1	0	0	1	1	M	Slp1 degradates Cdc13, that is inhibits complex Cdc2/Cdc13 and Cdc2/Cdc13*.
9	0	0	0	1	1	0	0	1	0	1	M	Antagonists of Cdc2/Cdc13 are reset.
10	0	0	0	1	1	0	0	1	0	0	G1	Cell reaches G1 stationary state (PP is inactive)

In the next step we run the model starting from each one of the 2^10^ = 1024 possible initial states. We find that each initial state flows into one of 13 stationary states (fixed points and one limit cycle). The largest attractor belongs to a fixed point attracting 73% of all network states. Our first observation is that this fixed point exactly coincides with the biological G1 stationary state (see Table 3) of the cell. Thus, the biological target state is the dominant attractor of the network dynamics. As soon as the system reaches this state with the specific corresponding combination of active and inactive proteins it stays there, and is likely to do so even in the presence of perturbations.

**Table 3:** All attractors (fixed points ( = FP) and one limit cycle ( = LC)) of the dynamics of the network model for the fission yeast cell cycle regulation.

Attractor	Type	Basin size	Start	SK	Cdc2/Cdc13	Ste9	Rum1	Slp1	Cdc2/Cdc13*	Wee1/Mik1	Cdc25	PP
1	FP	762	0	0	0	1	1	0	0	1	0	0
2	LC	208	0	0	0	0	0	0	0	0	1	1
	LC	0	0	0	0	0	0	1	0	0	1	0
	LC	0	0	0	1	1	1	0	1	1	0	0
3	FP	18	0	0	0	0	1	0	0	1	0	0
4	FP	18	0	0	0	1	0	0	0	1	0	0
5	FP	2	0	0	0	1	0	0	0	0	0	0
6	FP	2	0	0	0	1	0	0	0	0	1	0
7	FP	2	0	0	0	1	0	0	0	1	1	0
8	FP	2	0	0	0	0	1	0	0	0	0	0
9	FP	2	0	0	0	0	1	0	0	0	1	0
10	FP	2	0	0	0	0	1	0	0	1	1	0
11	FP	2	0	0	0	1	1	0	0	0	0	0
12	FP	2	0	0	0	1	1	0	0	0	1	0
13	FP	2	0	0	0	1	1	0	0	1	1	0

**Figure 2:**
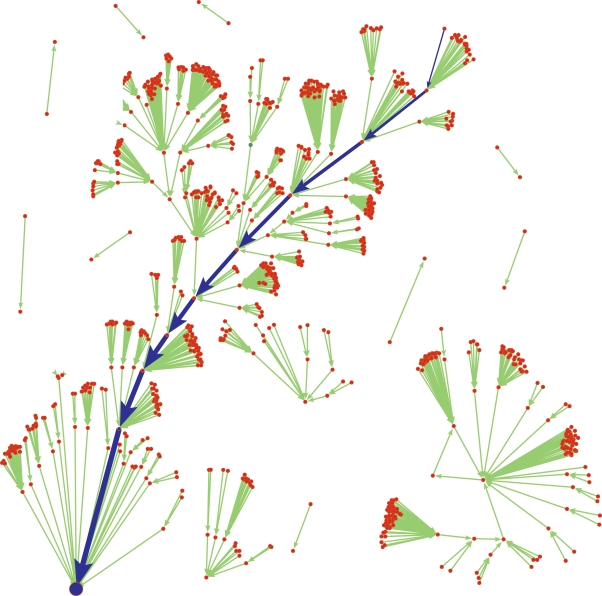
Network state space. State space of the 1024 possible network states (green circles) and their dynamical trajectories, all converging towards fixed point attractors. Each circle corresponds to one specific network state with each of the ten proteins being in one specific activation state (active/inactive). The largest attractor tree corresponds to all network states flowing to the G1 fixed point (blue node). Arrows between the network states indicate the direction of the dynamical flow from one network state to its subsequent state. The fission yeast cell-cycle sequence is shown with blue arrows.

A further observation is best depicted by Figure 2, showing the dynamical flow of the network states and how it converges towards the biological fixed point. In this figure, the dynamical trajectories in the state space starting from all 1024 possible initial states of the network are shown. Each network state is represented by a dot, with the arrows between them indicating the dynamical transition from one state to its temporally subsequent state. At the root of the largest attractor (tree) the G1 state is found and the blue arrows show the biological time sequence that leads to it. This attractor tree consists of 73% of all network states.

We additionally checked the probability of reaching the G1 stationary state, starting only from those initial conditions, when “Start” is active on the first time step. Here, in 75% of the cases one ends up in the G1 fixed point.

We further performed a robustness test by reversing the state of a single, randomly chosen node while the network proceeds through the biological sequence. This deviation from the biological pathway by the activity state of one single protein at one randomly chosen step of the cycle, the system returns to the fixed point G1 in 81 out of 100 possible cases. Thus we observe an additional robustness in the fission yeast cell-cycle network, meaning that there is an increased probability to stay in the attractor basin of the biological fixed point when perturbing states along the biological trajectory.

An immediate question about the specific network structure considered here is whether the architecture of the network has special properties as, for example, traces of being optimized by biological evolution. We compare the network dynamics to the null model of random networks with the same number of inhibiting and activating links, self-degrading and self-activating nodes and the same activation thresholds. Indeed one finds that the corresponding random networks typically have smaller attractors. The mean size of the biggest attractors is about 40% of all initial states (averaged over 1000 random networks). This may indicate that attractor basin size of the biological attractor is optimized to provide additional dynamical robustness.

### Comparison with *S. cerevisiae*

The two yeasts, *S. cerevisiae* and *S. pombe*, are remarkably different organisms and a comparison may provide insights relevant for the understanding of higher eukaryotes. As we now have discrete dynamical models for the cell cycle network of both of them at hand (this work, as well as [13]), let us discuss how they compare.

**Figure 3:**
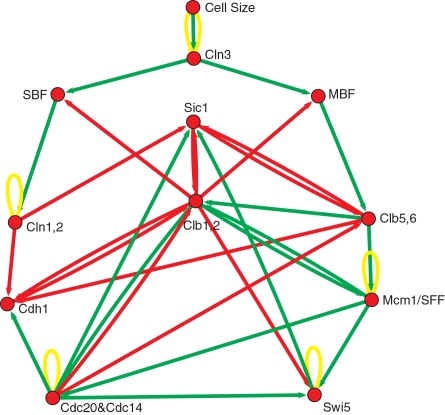
Budding yeast model. Budding yeast cell cycle network model [13] for comparison with our model of fission yeast. This network relies on transcriptional regulation more than the fission yeast network. Note that some homologues corresponding to the latter do not have to be included here. Note also the difference in circuitry.

As these two organisms are closely related genetically, one might expect a large overlap also in the biochemical control machinery. On the other hand, the biology of the two is markedly different, so there have to be some differences on the biochemical level as well. As an overview, the second model is shown in Figure 3.

There are a number of closely related genes (see Table 4) between the two yeasts [10], which, however, can have vastly differing functions [23]. In fission yeast, for example, phosphatase Cdc25 is required for the G2—M transition, while in the model of budding yeast [13] the corresponding homologue Mih1 is insignificant. The reason is that in the fission yeast cell cycle, Cdc25 removes an inhibitory phosphate group from the residue Tyr-15 of Cdc2, which is important for the right timing of the G2—M transition. In contrast, the tyrosine residue in *S. cerevisiae* Cdc28 kinase (fission yeast: Cdc2) is not as critical and usually not phosphorylated. Therefore, for a model of fission yeast, Cdc25 is essential, whereas the homologue Mih1 in budding yeast is not [13]. One other example is the role of the protein Cdc13. In fission yeast it acts in a complex with Cdc2, while in the budding yeast model its functionality is represented by two complexes Clb1,2/Cdc28 and Clb5,6/Cdc28, which exhibit some differences in interactions, as well as in timing.

**Table 4:** Homologue proteins related to the cell cycle networks of fission yeast and budding yeast.

Fission yeast	Rum1	Ste9	Slp1	Cdc2	Cdc13
Budding yeast	Sic1	Cdh11	Cdc20	Cdc28	Clb1-6

Despite of the differences in many details, the general logic of both yeast cell cycles is surprisingly similar and exhibits a number of “structural homologues”. For example, both exhibit a negative feedback loop similar in role: Clb1,2/Cdc28 activates Cdc20 which inhibits Clb1,2/Cdc28 (fission yeast: Cdc2/Cdc13 activates through Cdc25 Cdc2/Cdc13*, which activates Slp1, which in turn inhibits Cdc2/Cdc13, Cdc2/Cdc13*).

The most interesting comparison is in our view on the level of the global network dynamics. From this point of view, the S. cerevisiae network is a strongly damped system, driven by external excitation. External signals are entering the network, triggering signal cascades in the network that induce the subsequent phases. In contrast, the network of *S. pombe* corresponds to an auto-excited system (driven by a node with self-excitation-Cdc2/Cdc13) with additional damping. Here, an external signal works as a trigger mechanism that counteracts internal damping, causing the auto-excitation to spread its activity in the system

While these differences in the “mechanics” of the signaling networks are considerable, the overall dynamics is surprisingly similar. The state space picture is quite similar in both cases: one observes only a small number of attractors and just one big global attractor (with 86% resp. 73% of all initial states), which for both organisms corresponds to the stationary G1 state.

Finally, a most prominent difference between the two yeast networks is their choice in biochemical machinery: *S. cerevisiae* relies more on transcriptional factors while *S. pombe* mostly relies on post-translational regulation [35]. From the methodological point of view, we note that for this reason we were surprised to find our model for the *S. pombe* cell cycle network so robust against neglecting the vastly different time scales of interactions, which we expected to be the major difficulty in constructing a discrete dynamical model for *S. pombe* as compared to *S. cerevisiae*.

## Discussion

We have constructed a Boolean model for the biochemical network that controls the cell cycle progression in fission yeast *S. pombe*, and found a number of interesting results. The dynamics of this network reproduces the time sequence protein activation along the biological cell cycle, solely on the basis of the connectivity graph of the network, neglecting all biochemical kinetic parameters. The dynamics of the network is characterized by a dominant attractor in the space of all possible states, with an attractor basin that attracts most of all states. The network dynamics is robust against perturbation of the biological sequence of protein activation.

Also there is an interesting result, that the second big attractor is a limit cycle. This limit cycle could be related to the Wee1-Cdc25 double mutant. These cells have quantized cell cycles [9] as a result of an underlying oscillator that creates small amplitude oscillations in Cdc2/Cdc13 activity (with a role of Slp1 in this).

The overall results obtained from our model are in accordance with the existing ODE model of fission yeast [10]. Let us discuss the differences between these two approaches. The *S. pombe* ODE system [10] has several steady state solutions. One can identify every such solution with the corresponding physiological stage. The growth of cell size brings the cell from one phase to another via a series of bifurcations. At the same time, other variables indicate the degree of activity of various components of the cell regulatory nodes. One observes [26] that the typical curves depicting this activity have almost rectangular shape. This motivates our choice of binary valued function to approximate protein concentrations in time. Further, the ODE-based model makes use of continuous system parameters, which we omit and replace by their signs, only. As a result, the ODE bifurcation curve then corresponds to the Boolean biological path. The main advantage of our Boolean model is that we were able to drop 47 kinetic constants that were necessary in the ODE approach and, while doing so, still reproduce the biological sequence of protein activation.

This fact and our further observations point at built-in dynamical robustness of the network, which may provide a mechanism for organisms to ensure functional robustness [36]. Vice versa, our study indicates that the regulatory robustness of biological chemical networks may allow for “robust” modeling approaches: Our paradigm here is nothing but assuming that biochemical networks are functioning in a parameter-insensitive way—which motivated us to eliminate tunable parameters alltogether. That our model reproduces the biological sequence instantly without any further parameter tuning, confirms our assumption *a posteriori*. We therefore encourage further modeling experiments with the here presented, quite minimalistic approach as it may prove a quick route to predicting biologically relevant dynamical features of genetic and protein networks in the living cell.

## Materials and Methods

The network of the key regulators of the fission yeast cell cycle is constructed by compiling information from an extensive literature study [9], [25]–[34]. For building a model, all types of interactions are divided into two classes—inhibition or activation. The summarized interactions are shown in Table 1, which correspond to [9] except for the cases explained below.

Since the mechanism of activation of the negative Cdc2/Cdc13 regulators is unknown, the authors of [9] assumed a mechanism similar to budding yeast. In [9] Slp1/APC degrades a hypothetical inhibitor of PP, which helps PP to become active. Recently, Clp1p has been proposed as a possible candidate for PP [37]. Following [25], the helper molecules such as Start Kinases (SK) are inhibited, otherwise they prevent the final transition to the G1 stationary state. This is why in a Boolean model of the cell cycle helper molecules-Start Kinase (SK), Slp1, and PP-have self-inhibiting links. We further represent Wee1/Mik1 by one node, since they have similar function.

One also needs to distinguish activation levels of Cdc2/Cdc13. During the cell cycle, this complex has three different levels—low, intermediate, or high. It is also known that a high-level corresponds to dephosphorylation of the residue Tyr-15 of Cdc2. Therefore, Cdc2/Cdc13 is represented by two nodes: Cdc2/Cdc13 and Cdc2/Cdc13*, where the latter indicates the high activity state of Cdc2/Cdc13. During the G1 phase, when activity of Cdc2/Cdc13 is low, this corresponds to an inactive Cdc2/Cdc13 node. Intermediate levels of excitation correspond to activation of the node Cdc2/Cdc13, whereas high activity in the M phase is represented by the Cdc2/Cdc13* node being active in addition.

We focus on a case where checkpoints are disregarded except the checkpoint of the cell size. Also the change in the rate of DNA replication is neglected in the model. In comparison to [9] we further neglect the phosphatase group Pyp3, which works in the absence of Cdc25, but does its job less effectively.

The networks and dynamical trajectories were drawn with Pajek [38].
